# Low Cholesterol due to *APOB* Variants: Exploring the Balance Between Liver and Cardiovascular Risk

**DOI:** 10.1111/liv.70515

**Published:** 2026-01-19

**Authors:** Alessia Di Costanzo, Ilaria Pirona, Silvia Buonaiuto, Stella Covino, Carlo Maiorca, Simone Bini, Daniele Tramontano, Ilenia Minicocci, Francesco Baratta, Vincenza Colonna, Allegra Via, Laura D'Erasmo, Marcello Arca

**Affiliations:** ^1^ Department of Translational and Precision Medicine Sapienza University of Rome Rome Italy; ^2^ Istituto di Patologia Speciale Medica Catholic University of the Sacred Heart Rome Italy; ^3^ Department of Genetics, Genomics and Informatics University of Tennessee Health Science Center Memphis Tennessee USA; ^4^ Geriatric Unit, Department of Internal Medicine and Medical Specialties AOU Policlinico Umberto I Rome Italy; ^5^ Department of Biochemical Sciences “A. Rossi Fanelli” Sapienza University of Rome Rome Italy

**Keywords:** *APOB* gene, cardiovascular protection, drug targets, inactivating variants, liver disease risk

## Abstract

**Background & Aims:**

Lifelong *APOB* gene inactivation lowers LDL‐C and cardiovascular risk, but impairs hepatic lipoprotein export, predisposing to chronic liver disease (CLD). The extent to which common steatogenic factors modulate this risk remains unclear. Moreover, the balance between long‐term cardiovascular protection and CLD risk in *APOB* variant carriers has never been evaluated.

**Methods:**

Using UK Biobank data, we analysed 241 *APOB* loss‐of‐function (LoF) carriers and 410 721 non‐carriers, stratified by steatogenic risk factors, including age, sex, diabetes, BMI, alcohol intake and the *PNPLA3*‐rs738409 genotype. Associations with transaminase levels, CLD and cardiovascular (ASCVD) outcomes were assessed using Python and R packages.

**Results:**

*APOB* carriers had ~35% lower LDL‐C and apoB levels, along with reduced total triglycerides and Lp(a) (all *p* < 0.001). Baseline ALT and AST were higher in carriers than in non‐carriers (*P*_adj_ = 3.6 × 10^−7^), particularly among those with obesity (*p* ≤ 0.003). The prevalence and incidence of CLD were consistently higher in carriers across all risk factor categories (*p* ≤ 0.01), with the strongest association in those with diabetes and obesity over 15 years of follow‐up (*P*_adj_ = 0.03). In contrast, *APOB* carriers as a whole had a 57% lower ASCVD risk (*P*_adj_ *=* 0.009), with a similar atheroprotective trend across all risk factor categories. This corresponded to an absolute risk reduction of 2.30 ASCVD events/1000 person‐years (*p* = 0.002) and an absolute increase of 3.48 CLD events/1000 person‐years (*p* = 0.003).

**Conclusions:**

Long‐term exposure to low LDL‐C levels due to *APOB* LoF variants has opposite consequences, reducing ASCVD risk but increasing CLD risk, especially in the presence of diabetes and obesity. These findings highlight the importance of balancing cardiovascular benefit with hepatic safety when considering apoB‐targeting therapies.

## Lay Summary

- Genetically determined inhibition of apoB has opposite long‐term clinical consequences, revealing a trade‐off between cardiovascular protection and increased liver disease risk, particularly in individuals with diabetes or obesity.
- When considering therapies targeting the apoB pathway, metabolic risk profiling and liver safety evaluation should be integrated into clinical decision‐making, alongside assessment of cardiovascular benefit.

**Abbreviations**
ALT: alanine aminotransferase
*APOB*
: apolipoprotein B gene
ARD: absolute risk difference
ASCVD: atherosclerotic cardiovascular disease
AST: aspartate aminotransferase
BMI: Body mass index
CLD: chronic liver disease
FHBL1: familial hypobetalipoproteinemia type 1
ICD: International Classification of Diseases
IRs: incidence rates
LDL‐C: low‐density lipoprotein cholesterol
LoF: loss‐of‐function
NCB: net clinical benefit
PCA: principal components of ancestry
PY: person‐years
T2D: type 2 diabetes
UKBB: UK Biobank
VLDL: very low‐density lipoproteins

## 1 Introduction

In recent years, much pharmacological research has been devoted to identifying low‐density (LDL)‐lowering drugs acting by mechanisms not involving the activation of LDL receptor (LDLR) pathway but rather by reducing very low‐density lipoproteins (VLDL) secretion [1]. While any reduction in LDL‐C lowers the risk of atherosclerotic cardiovascular disease (ASCVD) [2], the long‐term safety of achieving these reductions via pharmacological blockade of hepatic VLDL synthesis represents a matter of debate [3].

One way to address this question might be to study individuals carrying loss‐of‐function (LoF) variants in drug target genes [4, 5, 6]. In this regard, an interesting model is represented by patients affected by familial hypobetalipoproteinemia type 1 (FHBL1) who manifest a low LDL‐C status as a consequence of the presence of inactivating variants in the *APOB* gene [7]. Nevertheless, this condition is associated with impaired synthesis and export of VLDL by the liver [8] which, in turn, causes liver steatosis that may eventually progress towards cirrhosis and, even, hepatocellular carcinoma (HCC) [9].

Indeed, in a large‐scale investigation using data from the UK Biobank (UKBB), we recently demonstrated that the genetic inactivation of *APOB* confers an about 4‐fold increased life‐long risk of chronic liver disease (CLD) [10]. Nevertheless, the impact of metabolic risk factors on this association has not been fully explored. More importantly, it has never fully estimated whether in these patients the potential health benefit resulting from cardiovascular protection is somehow limited by the presence of an increased risk of CLD. We believe that these aspects are important for understanding the cost–benefit ratio of current and future therapeutic strategies targeting the synthesis or secretion of VLDL with the aim of improving the cardiovascular risk.

Based on these premises, the objective of this study was threefold. Firstly, we investigated the association between *APOB* inactivating variants and early markers of liver injury, that is, serum transaminases, after stratifying *APOB* carriers and non‐carriers by genetic and non‐genetic steatogenic risk factors. Secondly, we evaluated the potential deleterious effects of these steatogenic risk factors on the composite outcome of CLD, considering the prevalence risk as well as the lifetime and cumulative incidence. Finally, we assessed the impact of *APOB* inactivating variants on the risk of ASCVD, providing an indirect yet balanced assessment of the benefits and potential drawbacks associated with the *APOB*‐linked low LDL‐C phenotype.

## 2 Material and Methods

### 2.1 Study Population, Setting and Design

The UKBB is a prospective cohort study that enrolled approximately 500 000 individuals across the United Kingdom between 2006 and 2018 whose characteristics have been detailed elsewhere [11]. A sample of 410 962 participants were included in the present analysis and the flowchart for enrolment is shown in the Figure S1.

The Northwest Multi‐Center Research Ethics Committee approved the UKBB study, and all study participants provided written informed consent. Individuals who had revoked consent were excluded from the present analysis.

### 2.2 Selection of Inactivating Variants in *APOB* and Ascertainment of Their LDL‐Lowering Effect

We used data from the genotyping array (500 000 participants) and whole‐exome sequencing (250 000 individuals) to search for loss‐of‐function (LoF) variants in the *APOB* gene. We performed a genotype concordance check, finding no differences between the two methods.

Rare variants (MAF < 0.1%) were classified as LoF if they predicted premature protein truncation, insertions or deletions and point mutations in the splicing site [5, 12]. Further details for selecting *APOB* variants and determining their LDL‐C lowering effect are provided in Supporting Information [13, 14, 15, 16, 17, 18, 19].

### 2.3 Assessment of Individual Characteristics

Clinical and biochemical characteristics of study individuals collected at the time of their enrollment into the UKBB were retrieved. Hypertension and type 2 diabetes (T2D) were diagnosed by using the International Classification of Diseases (ICD) Ninth and Tenth revisions (ICD‐9 and ICD‐10) codes as listed in the Table S1. Body mass index (BMI) was categorised as normal (BMI < 25 kg/m^2^), overweight (25 ≥ BMI < 30 kg/m^2^) and obese (BMI ≥ 30 kg/m^2^) [20]. To evaluate alcohol consumption, all patients with no reported alcohol intake were classified as non‐drinkers (*n* = 31 417), and all the others as drinkers (*n* = 379 545). To consider potential genetic susceptibility to liver diseases, the patatin‐like phospholipase domain‐containing protein 3 (*PNPLA3*—rs738409, chr:22) and the transmembrane 6 superfamily 2 (*TM6SF2*—rs58542926, chr:19) genotypes were retrieved and used as covariates in the statistical analyses. We accounted for the effect of lipid‐lowering therapies use at the time of lipid measurements by dividing the measured total cholesterol (TC) and LDL‐C by 0.8 and 0.7, respectively [6]; HDL‐C and TG values were not adjusted.

### 2.4 Study Outcomes

Changes in plasma levels of liver transaminases (ALT and AST) and the incidence of CLD were used to estimate liver outcomes. Chronic liver disease was defined as the composite of alcoholic and non‐alcoholic fatty liver, liver cirrhosis and liver cancer [18, 21]. ICD codes were used to determine the primary and secondary causes of liver‐related deaths (Table S1). ASCVD cases were defined as those having at hospital discharge diagnosis of ischemic heart disease, myocardial infarction and complications of heart disease (see Table S1). For both liver and ASCVD outcomes, only the first diagnosis reported in the database was counted. Additional details are provided in Supporting Information.

### 2.5 Statistical Analysis

Normality was assessed using the Kolmogorov–Smirnov test. Non‐normally distributed variables were compared using the Mann–Whitney test, while categorical traits were compared using the chi‐square test. Analyses were restricted to individuals with complete data (no imputation or exclusion of individuals with missing data), and only differences in missingness > 3% between groups were reported in the tables.

Associations between high‐confidence LoF variants and lipid traits were tested using mixed linear models (Python statsmodels‐scipy [22]) adjusting for age, age^2^, sex, the first 10 principal components of ancestry (PCA) and UKBB assessment centres. Analysis of liver enzymes was stratified by tertiles of risk factors (age, sex, BMI categories, T2D status, alcohol consumption, categorised as yes or never and *PNPLA3* rs738409 genotypes, assuming a dominant model). Age and BMI were modelled as ordered categorical variables (1–3) [20]. For stratified analyses, *TM6SF2* rs58592626 was excluded due to low *APOB* carrier numbers (*n* = 37).

Logistic and Cox regression models were used to estimate odds ratios (ORs) and hazard ratios (HRs) for CLD and ASCVD risk, including event‐free survival and cumulative incidence over 15 years of follow‐up. Participants were censored at death, loss to follow‐up or at the end of data collection in May 2022, which corresponded to the date of data receipt (mean follow‐up: 12 ± 2.1 years). In the 15‐year COX analysis, all individuals with pre‐existing disease at baseline were excluded.

Overall, CLD risk was estimated after adjustment for age, sex, BMI, T2D, alcohol intake, *PNPLA3* rs738409 and *TM6SF2* rs58592626 genotypes, while ASCVD risk was estimated using two models: Model 1 (age, sex, hypertension, T2D, smoking, first 10 PCA) and Model 2 (Model 1 + estimated untreated LDL‐C levels). Additional adjustments were performed by including total triglycerides, HDL or ApoA‐I as covariates, individually.

Risk prevalence and incidence of CLD and ASCVD outcomes were further analysed after stratifying for risk factors. When subgroup analyses resulted in extremely small groups or very few events, we applied Firth's penalised likelihood method to reduce small‐sample bias and bootstrap resampling (1000 iterations) to evaluate the stability and robustness of the effect estimates.

Incidence rates (IRs) of CLD and ASCVD were calculated by dividing the number of individuals with events by the corresponding total number of person‐years (PY) of follow‐up in each group. The absolute risk difference (ARD) was evaluated by subtracting the incidence rate in *APOB* carriers from that in noncarriers for each outcome. Finally, the Net Clinical Benefit (NCB) was defined as follows, assuming equal weighting (W) for both outcomes: NCB = (IR_ASCVD,noncarriers_ − IR_ASCVD,*APOB*carriers_) − W × (IR _CLD,*APOB* carriers_ − IR_CLD,noncarriers_) [23].

All analyses were performed using Python v3.10.13 and R v4.3.1. Two‐tailed *p* values ≤ 0.05 were considered as statistically significant.

## 3 Results

### 3.1 Clinical and Biochemical Characteristics of Study Groups

Demographic and clinical characteristics of individuals according to the *APOB* mutational status are provided in Table 1. The groups did not differ in age, BMI and sex distribution; however, carriers of *APOB* variants showed a 38.4% reduction in LDL‐C (*p* = 9.9 × 10^−72^) and a 33% reduction in ApoB (*p* = 5.2 × 10^−38^). These results were confirmed even after adjustment for age, sex, age^2^ and the first 10 principal components of ancestry (PCA). Figure S2 shows the distribution of LDL‐C levels according to each *APOB* variant compared to non‐carriers. Additionally, *APOB* carriers showed lower Lp(a) levels (*p* < 0.001) and plasma total triglycerides (TG) (*p* = 2.2 × 10^−20^).

**TABLE 1:** Demographic and clinical characteristics of individuals according to the mutational status.

	Non‐carriers *n* = 410 721	APOB carriers *n* = 241	*p*
Age, years	57.0 (49–62)	57.0 (51–63)	—
Sex (male/female), *n*	177 424/233297	88/153	—
Smoking (current), *n* (%)	41 756 (10.2)	24 (9.9)	—
Alcohol consumption, (never)	31 390 (7.6)	27 (11.2)	0.03
Self‐reported ethnicity, *n* (%)			
White	387 732 (94)	223 (96)	—
Black	2803 (0.7)	0	—
Asian	7046 (1.7)	6 (2.4)	—
Chinese	1372 (0.3)	2 (0.8)	—
Mixed background	2527 (0.6)	1 (0.4)	—
Other or not reported	8560 (2.1)	8 (3.3)	—
Medications			
Lipid‐lowering Medications, *n* (%)	44 260 (10.7)	8 (3.3)	< 0.001
Anti‐hypertensive therapy, *n* (%)	65 847 (16)	54 (22.4)	< 0.01
Cormobidity			
Obesity (BMI ≥ 30 kg/m^2^), *n* (%)	92 171 (22.4)	65 (26.5)	—
Diabetes, *n* (%)	21 200 (5)	25 (10.3)	0.0002
Hypertension, *n* (%)	87 770 (21.4)	70 (29)	< 0.01
Anthropometric data			
Body mass index (BMI) kg/m^2^	26.5 (23.96–29.59)	26.23 (24.04–30.19)	—
Waist Circumference (cm)	89.0 (80.0–98.0)	88.0 (80.0–98.0)	—
Systolic blood pressure, mmHg	137.0 (125.0–151.0)	138.0 (126.0–151.0)	—
Diastolic blood pressure, mmHg	82.0 (75.0–89.0)	82.0 (75.0–89.0)	—
Lipid concentrations			
Total cholesterol, mg/dL	222.15 (197.64–249.09)	163.21 (132.98–194.62)	4 × 10^−66^
LDL‐C, mg/dL	140.37 (121.49–161.45)	86.45 (65.17–118.24)	9.9 × 10 ^−72^
HDL‐C, mg/dL	54.0 (45.33–64.45)	56.16 (46.7–67.49)	0.03
Total triglycerides, mg/dL	126.51 (89.87–182.44)	87.09 (59.99–136.33)	2.2 × 10^−20^
ApoB, g/L^a^	1.03 (0.89–1.19)	0.69 (0.51–0.94)	5.2 × 10^−38^
Blood glucose, mmol/L	4.9 (4.59–5.28)	4.9 (4.6–5.4)	—
Liver makers			
AST, U/L	24.1 (20.8–28.5)	25.2 (21.7–31.0)	0.001
ALT, U/L	19.7 (15.14–26.82)	22.1 (16.1–33.6)	0.0003
γ‐GT, U/L	25.4 (18.0–39.3)	26.8 (18.7–44.6)	—
Total—bilirubin, μmol/L	8.05 (6.42–10.49)	7.77 (6.32–10.26)	—
Direct—bilirubin, μmol/L	1.61 (1.30–2.06)	1.83 (1.43–2.50)	< 0.001
Albumin, g/L	45.25 (43.56–46.96)	45.38 (43.33–46.98)	—
Other biochemical measurements			
Apolipoprotein A‐I, g/L	1.52 (1.4–1.7)	1.54 (1.4–1.7)	—
Lp(a), mg/dL^b^	20.90 (9.6–61.3)	12.97 (7.3–51.1)	< 0.001
HbA1c, mmol/mol	35.0 (32.5–37.5)	36.2 (33.7–39.2)	< 0.001
C‐reactive protein, mg/L	1.28 (0.63–2.66)	1.89 (0.83–3.49)	< 0.001
Creatinine, μmol/L	69.8 (61.1–80.2)	69.8 (62.0–79.1)	—
Total—protein, g/L	72.34 (69.80–75.06)	72.48 (70.06–74.85)	—
Vitamin D, nmol/L^c^	46.9 (32.5–62.3)	54.2 (32.92–74.05)	< 0.001
Pro‐steatogenic gene variants			
*PNPLA3* rs738409 (CG + GG), *n* (%)^d^	153 764 (37)	78 (32)	0.05
*TM6SF2* rs58542926 (CT + TT), *n* (%)^e^	57 040 (14)	37 (15)	—

*Note:* Summary of baseline characteristics of individuals studied in the UK Biobank. Columns show participants grouped according to carrier status. Rows show measurements at the initial UK Biobank assessment visit, with values corresponding to number (%) or median [25‐75th]. SI conversion factors: To convert cholesterol to mmol/L, multiply by 0.0259; triglycerides to mmol/L, multiply by 0.0113 [5, 6]. Variables with > 3% difference in proportion of missing data between compared groups are indicated: ^a^Baseline apoB levels were reported for 382 363 to 410 721 individuals in the non‐carriers group, 169 to 241 individuals in the *APOB* group. ^b^Baseline Lp(a) levels were reported for 308 934 to 410 721 individuals in the non‐carriers group, 172 to 241 individuals in the *APOB* group. ^c^Baseline Vitamin D were reported for 366 680 to 410 721 individuals in the non‐carriers group, 222 to 241 individuals in the *APOB* group. ^d^ *PNPLA3* ra738409 genotypes were reported for 398 613 to 410 721 individuals in the non‐carriers group. ^e^ *TM6SF2* rs58592623 genotypes were reported for 398 999 to 410 721 individuals in the non‐carriers group. *p* values correspond to unadjusted comparisons between groups, as assessed by the chi‐squared test or Mann–Whitney test. Only significant comparisons are reported.

Abbreviations: ALT, alanine aminotransferase; AST indicates aspartate aminotransferase; GGT indicates gamma glutamyl transferase.; HDL indicates high‐density lipoprotein; LDL, low‐density lipoprotein; Lp(a) indicates lipoprotein a.

Interestingly, the prevalence of T2D was higher in carriers than non‐carriers (*p* = 0.0002). This difference remained significant after adjustments for confounders (all *P*_adj_ < 0.001).

Among the available biochemical markers, ALT and AST were significantly higher in *APOB* variant carriers compared with non‐carriers (*p* = 0.0024 for ALT; and *p* = 0.0075 for AST), whereas γ‐GT, albumin and bilirubin levels did not differ between groups. These differences remained highly significant even after adjustment for age, sex, BMI, T2D, alcohol consumption and *PNPLA3* genotype (all *P*_adj_ ≤ 8.1 × 10^−6^). Notably, AST and ALT levels were strongly correlated in both *APOB* carriers and non‐carriers (Spearman *r* = 0.76 and *r* = 0.65, respectively; both *p* < 0.001), indicating that individuals with elevated AST also exhibited elevated ALT.

### 3.2 Changes in Liver Enzymes According to *APOB* Variants Stratified by Steatogenic Risk Factors

**FIGURE 1:**
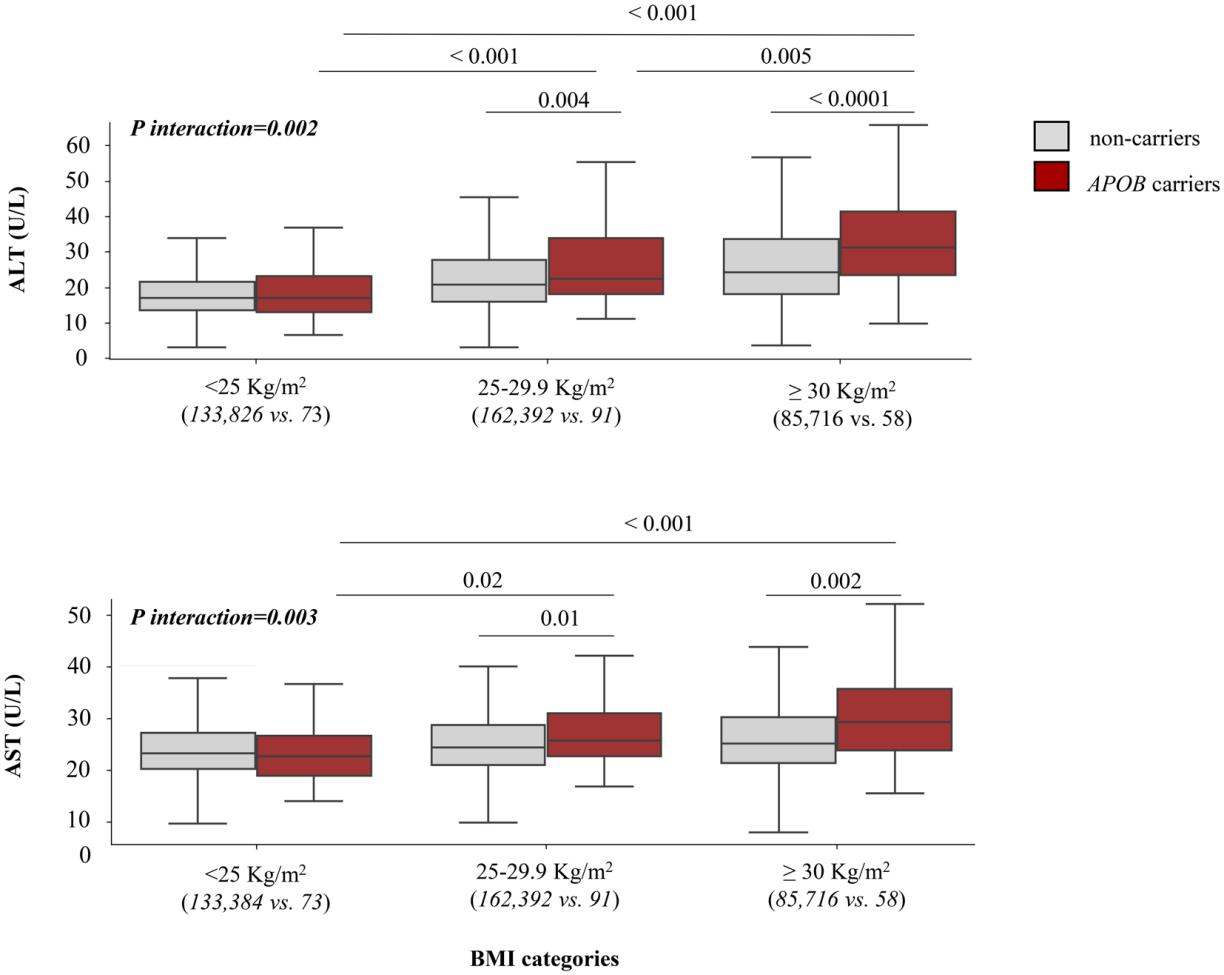
Obesity modulates the effect of *APOB* inactivating variants on liver transaminase elevation. Serum levels of ALT and AST according to *APOB* carrier status and BMI categories. Bars represent mean transaminase levels (U/L) and 95% CI. BMI categories: Normal weight (T1: < 25 kg/m^2^), overweight (T2: 25–29.9 kg/m^2^) and obese (T3: ≥ 30 kg/m^2^). Only Significant *p*‐values are reported. The number of carriers and non‐carriers are reported for each subgroup analysis. ALT and AST levels were available for 381 949 to 410 721 individuals in the non‐carrier group, 226 to 241 individuals in the *APOB* group. ALT, alanine aminotransferase; AST, aspartate aminotransferase.

After stratification for potential pro‐steatogenic risk factors, obesity enhanced the effect of *APOB* variants on ALT and AST levels (Figure 1). This was confirmed by a significant interaction effect between *APOB* variants and BMI (*P interaction* = 0.002 for ALT; *P interaction* = 0.003 for AST). In contrast, no significant additive or interaction effects were observed between *APOB* variants and age, sex, T2D status, *PNPLA3* genotypes or alcohol consumption on ALT and AST levels (data not shown). Additional stratified analyses including γ‐GT, albumin and bilirubin levels did not reveal any significant associations or interactions with steatogenic risk factors within the *APOB* group, nor when compared with non‐carriers.

### 3.3 Risk of CLD in *APOB* Carriers Stratified by Steatogenic Risk Factors

Among the 410 787 UKBB individuals included in the analysis, 5967 were affected by liver‐related disease: 16 of 241 *APOB* carriers and 5951 of 410 546 non‐carriers. This translated into an overall 4‐fold increased risk of CLD in *APOB* carriers as compared to non‐carriers (*P*_adj_ = 3.3 × 10^−8^). Similarly, the risk of death from CLD was 7‐fold higher in the *APOB* group than in non‐carriers, even after adjustments for confounders (OR 7.10, 95% CI 1.70–29.63, *P*_adj_ = 0.007).

Given that age (OR 1.04, 95% CI 1.00–1.10, *p* = 0.05), sex (OR 1.26, 95% CI 1.20–1.33, *p* < 0.001), BMI (OR 1.07, 95% CI 1.06–1.07, *p* < 0.001), T2D (OR 4.17, 95% CI 3.89–4.46, *p* < 0.001), alcohol intake (OR 2.92, 95% CI 1.00–9.82, *p* < 0.001) and *PNPLA3* rs738409 genotypes (OR 1.37, 95% CI 1.32–1.43, *p* < 0.001) associated with the risk of prevalent CLD, we stratified the analysis according to these risk factors.

**FIGURE 2:**
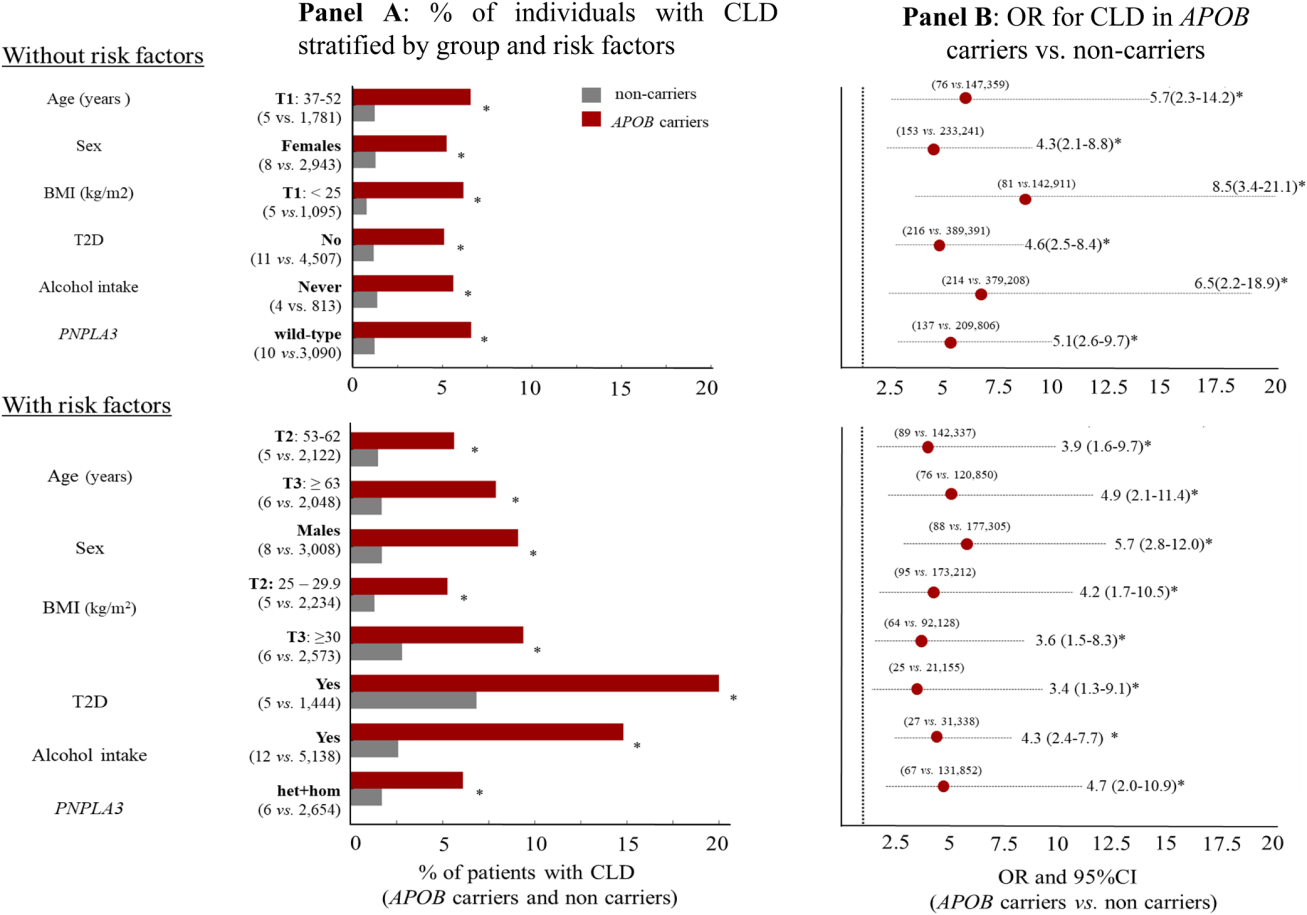
*APOB*, steatogenic risk factors and risk of chronic liver disease (CLD) in the UKBB cohort. Panel A shows the percentage of individuals with CLD stratified by the presence or absence of risk factors, while Panel B displays the odds ratios (ORs) and 95% confidence intervals (CIs) for CLD in *APOB* carriers versus non‐carriers. The numbers of carriers and non‐carriers are reported for each subgroup analysis. * all *p* < 0.001 for comparisons between *APOB* carriers and non‐carriers; *p* = 0.0004 for comparison between *APOB* carriers with and without T2D (Panel A); *p* = 0.008 for the same comparison in Panel B. Firth‐penalised regression and bootstrapping were applied (see Material and Methods).

Although both prevalence and risk of CLD (Figure 2, Panel A and Panel B) were higher in *APOB* variant carriers than in non‐carriers across all risk categories (all *p* ≤ 0.01), the most pronounced difference was observed in individuals with T2D. This generated a 4.6‐folder higher risk of prevalent CLD in *APOB* carriers with T2D (*n = 25*) compared to carriers without T2D (*n* = 216) (OR 4.6, 95% CI 1.47–14.7, *p* = 0.008). Similar results were obtained when comparing lifetime event‐free survival rate between *APOB* carriers with T2D and those without T2D (HR 4.2, 95% CI: 1.41–12.77, *p* = 0.009). By contrast, no significant additive nor interaction effect of age, sex, BMI, alcohol consumption or the *PNPLA3* rs738409 genotype was observed.

These results were reinforced when we calculated the lifetime risk of CLD in *APOB* carriers without T2D and with normal BMI (BMI < 25 kg/m^2^, *n* = 80) compared to *APOB* carriers with both T2D and obesity (BMI ≥ 25 kg/m^2^, *n* = 23). The latter group exhibited a 4‐fold higher lifetime risk of CLD events (HR 4.0, 95% CI 1.08–15.10, *P*_adj_ *= 0.03*), which remained significant after adjustment for age and sex (HR 5.5, 95% CI 1.33–22.93, *P*_adj_ *= 0.012*). Similar results were observed when comparing the cumulative incidence of CLD events within 15 years of follow‐up between the *APOB* carriers without T2D and obesity (*n* = 80) vs. those with both T2D and obesity (*n* = 22) (HR 4.8, 95% CI 1.08–21.9, *P*_adj_ *= 0.05* adjusted for age and sex).

### 3.4 *APOB* Inactivating Variants and Risk of ASCVD

Among the 410 962 individuals considered in the present analysis, 30 797 were classified as affected by ASCVD. After adjusting for confounding factors, *APOB* carriers showed a 57% lower risk of developing ASCVD compared to non‐carriers (OR 0.43, 95% CI 0.23–0.81; *P*_adj_ = 0.009).

**FIGURE 3:**
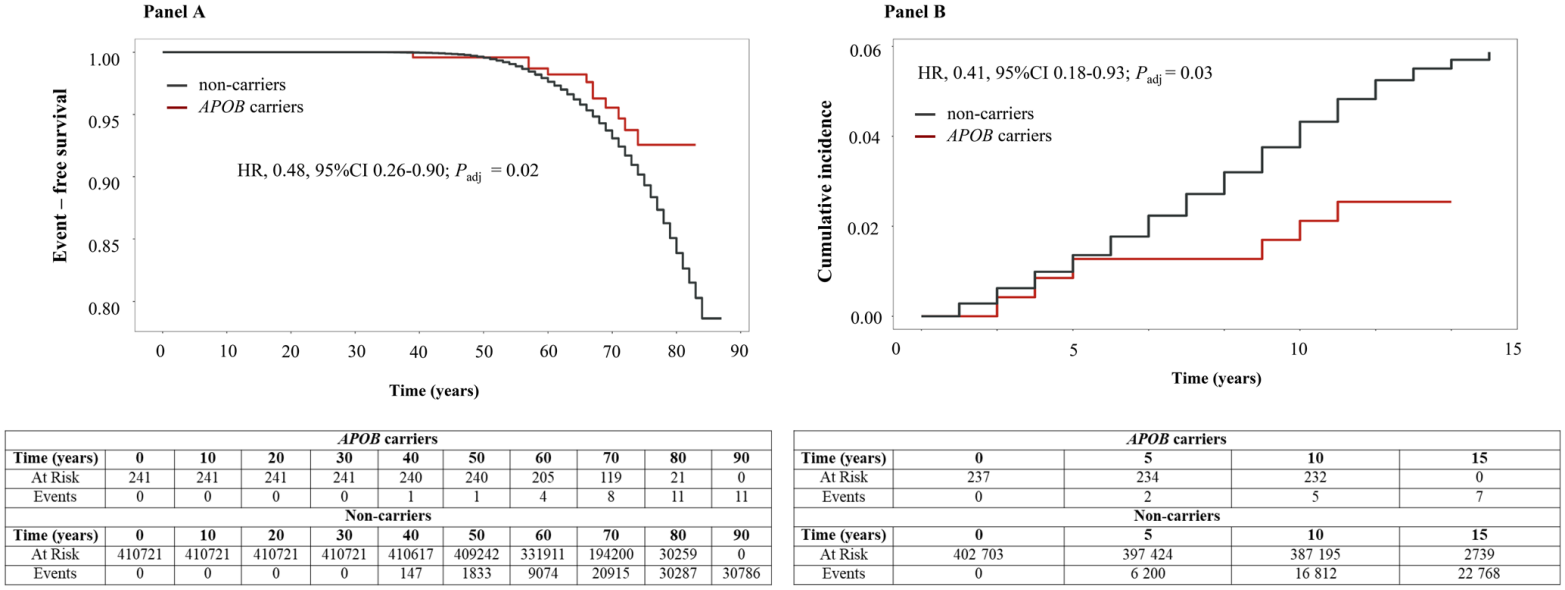
Risk of ASCVD events according to *APOB* carrier status. Kaplan–Meier curves and multivariable Cox proportional hazards regression models with covariates were used to estimate the incidence of ASCVD events between *APOB* carriers and non‐carriers. ASCVD was defined as reported in Supporting Information. Participant were followed until death or end of follow‐up in May 2022. Panel A: Event‐free survival. Panel B: Cumulative incidence over 15 years of follow‐up. All individuals with prevalent ASCVD in any group at enrolment were excluded. Curves were adjusted for age, sex, 10 PCA, smoking status, comorbidity (hypertension, diabetes) (Model 1). Additional adjustments including LDL‐C, total triglycerides, HDL or ApoA‐I as covariates, individually did not change the associations.

Figure 3, Panel A and Panel B illustrate the lifetime event‐free survival rate and the 15‐year incidence of ASCVD in *APOB* carriers compared to non‐carriers. *APOB* carriers show 52% lower lifetime incidence of ASCVD events (HR 0.48, 95% CI 0.26–0.90; *P*_adj_ = 0.006) and 59% lower risk of ASCVD events over a 15‐year follow‐up period (HR 0.41, 95% CI 0.18–0.93; *P*_adj_ < 0.001). A similar, reduced 15‐year ASCVD risk associated with the presence of *APOB* variants was detected in each age tertile, sex, obesity category, presence or absence of T2D, lipid tertiles (LDL‐C, HDL‐C, total triglycerides), apolipoprotein AI (apoAI) tertiles, alcohol consumption and *PNPLA3* rs738409 genotypes (see Figure S3). For many of these associations, HRs figures did not reach formal statistical significance mainly due to the small number of subjects included in each subgroup. However, these results were corrected by using the Firth penalization and/or bootstrap resampling providing similar trends thus further supporting the stability of the findings. To note, no significant additive or interaction effects of the evaluated risk factors were observed within the *APOB* group.

### 3.5 Divergent Effect of *APOB* Defective Alleles on the Incidence of CLD and ASCVD Risk

**FIGURE 4:**
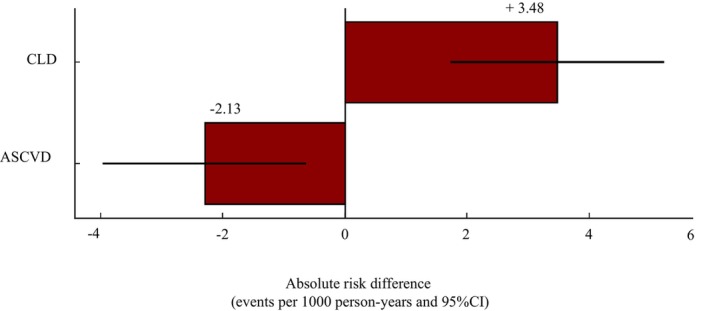
Absolute risk differences for ASCVD and CLD in *APOB* variant carriers vs. non‐carriers. Bar plot shows the absolute risk differences (ARD) of number of events per 1000 person‐years for atherosclerotic cardiovascular disease (ASCVD) and chronic liver disease (CLD), comparing individuals carrying APOB variants (exposed) versus non‐carriers (unexposed). Error bars represent 95% confidence intervals (CI).

We additionally calculated the incidence rates (IR) of both ASCVD and CLD, expressed as events per 1000 person‐years (PY). Among *APOB* variant carriers, the incidence of ASCVD was 2.08 per 1000 PY, compared to 4.38 per 1000 PY in non‐carriers (*p* = 0.002), resulting in an absolute reduction of 2.30 events per 1000 PY. In contrast, the incidence of hepatic events was 4.48 per 1000 PY in the *APOB* group compared to 0.99 per 1000 PY in non‐carriers (*p* = 0.003), resulting in an absolute increase of 3.48 events per 1000 PY (Figure 4). Assuming both outcomes carried equal weight, the NCB was approximately −1.19 per 1000 PY, indicating that the increase in hepatic risk outweighed the cardiovascular benefit. These results were confirmed after considering the cause of specific mortality, expressed as deaths per 1000 PY. *APOB* carriers had 0.060 ASCVD deaths per 1000 PY compared to 0.680 CLD deaths per 1000 PY.

## 4 Discussion

In this study, rare naturally occurring DNA variants that inactivate *APOB* were used to investigate the association between the lifelong exposure to lower LDL‐C levels and CLD and ASCVD risk, stratified by metabolic risk factors. Leveraging data from the UK Biobank (UKBB), we assembled a large cohort of 241 individuals carrying rare inactivating variants in the *APOB* gene who exhibited an approximate 35% reduction in LDL‐C, triglycerides and circulating apoB.

Using serum transaminases as surrogate markers of liver injury, we observed that *APOB* variant carriers exhibited mildly elevated ALT and AST levels compared to non‐carriers, and this association was particularly evident among obese carriers. These findings, which confirm previous reports [23, 24, 25], highlight the importance of weight management as a preventive strategy in *APOB* variant carriers to prevent liver complications.

Extending our previous observations [10, 25], we found that the excess of CLD among *APOB* variant carriers was independent from age, sex, BMI, diabetes, alcohol intake and *PNPLA3* genotype status. Notably, this association remained robust over 15 years of follow‐up, thus reinforcing the notion that impairing hepatic VLDL export alone may drive CLD risk. ApoB is crucial for proper assembly and secretion of VLDL by hepatocytes, and its inactivation results in hepatic TG retention [6]. Prior studies report a 74% reduction in VLDL apoB‐100 secretion in carriers [6, 7]. Although the mechanistic link between TG accumulation and CLD is unclear, it may involve inflammatory responses to lipid overload in combination with genetic predisposition [26, 27, 28].

In sensitivity analyses, we found that diabetes increased the risk of new‐onset CLD events by 4.8‐fold in *APOB* carriers, and this effect was further amplified by the presence of concurrent obesity. In our cohort, the prevalence of diabetes was 10% among carriers compared to 5% in non‐carriers. However, large case series and meta‐analyses have not supported a significant increase in diabetes among individuals with FHBL1 [29], concluding that genetically low LDL‐C due to *APOB* truncating variants is not inherently diabetogenic. Notably, in our cohort, the risk of CLD remained high in carriers without diabetes, suggesting that diabetes acts as an amplifier of CLD risk in *APOB* carriers. In contrast to a previous study [30], we found no significant interaction between *APOB* and *PNPLA3* rs738409 in favouring liver complications. This result was somewhat unexpected, given that *APOB* and *PNPLA3* variants are both linked to hepatic TG accumulation [31, 32]. One possible explanation is that the mechanistic effect of *APOB* variants may outweigh that of the *PNPLA3 rs738409* genotype.

Finally, we assessed the balance between the impact of *APOB* variants on liver and cardiovascular health. In line with previous studies [5, 12], *APOB* variants offered strong signals of protection against ASCVD, which were not offset by metabolic risk factors. Given that circulating ApoB‐containing lipoproteins are well‐established causal mediators of atherosclerosis, our results are biologically plausible. However, when we estimated the trade‐off between cardiovascular protection and the risk of liver complications, the latter significantly exceeded the degree of cardiovascular benefit conferred by low LDL‐C levels in this specific population. To our knowledge, no other similar studies are available in the literature; therefore, it is very difficult to compare our data to those of other investigations. In any case, it should be noted that the magnitude and direction of this balance cannot be generalizable to all LDL‐lowering interventions or to high‐risk patients' populations (i.e., those with homozygous FH), in whom the benefits of ApoB‐pathway inactivation may outweigh potential hepatic liabilities. Nonetheless, these findings emphasise the need for a personalised risk–benefit assessment when therapeutically targeting the ApoB pathway.

The strengths and limitations of the present study should also be considered. A major strength is the extended follow‐up period, which captured long‐term outcomes and enabled us, for the first time, to characterise the sustained impact of *APOB* inactivation over time on both hepatic and cardiovascular outcomes. To our knowledge, no previous cohort of *APOB* variant carriers or patients treated with drugs targeting the ApoB pathway (e.g., lomitapide) has provided comparable longitudinal insight [33, 34, 35]. Furthermore, we examined the full spectrum of ischemic and liver‐related outcomes, offering a detailed categorisation of clinical events. Limitations include the relatively small number of liver‐specific events, particularly cases of cirrhosis and HCC. In addition, liver function could not be comprehensively assessed; additional markers (e.g., albumin, bilirubin, γ‐GT or fibrosis scores) and imaging would have strengthened the analysis. Moreover, some of the subgroup analyses involved small sample sizes; however, we applied bias‐reduction methods which consistently confirmed the direction and significance of the results. Finally, we assessed the overall risk–benefit balance of carrying inactivating *APOB* variants using a single quantitative measure—the net clinical benefit—defined as the difference between the number of major adverse cardiac events avoided and the price to pay in terms of excess liver events. Although this approach has been criticised as misleading [36], it has been often used in other therapeutic contexts [23]. Finally, these findings are derived from a general population cohort composed predominantly of healthy individuals, as represented by the UKBB cohort [11].

In conclusion, our study provides a unique insight into the opposite long‐term clinical consequences of ApoB inactivation. Although carriers of LoF *APOB* variants exhibited a substantially reduced risk of atherosclerotic events, this benefit was outweighed by a significant long‐term increase in the risk of CLD, amplified in individuals with obesity or diabetes. In the context of emerging therapeutic strategies targeting the ApoB pathway, the meticulous monitoring of metabolic profiling in relation to potential hepatic complications is crucial to mitigate the potential hepatic burden while preserving the cardiovascular benefits.

## Author Contributions

Conception and design: A.D.C. and M.A. Acquisition of data, analysis or interpretation of data: A.D.C., I.P., M.A., L.D., V.C., A.V., S.B., F.B. Drafting the article: A.D.C., I.P., M.A., L.D. Revising it critically for important intellectual content: V.C., A.V., S.B., F.B., S.C., I.M., S.B., D.T., C.M., A.D.C. and M.A. Statistical analysis: A.D.C, P.I, S.B. All authors contributed to reviewing and approving the final version of the manuscript.

## Funding

The authors have nothing to report.

## Conflicts of Interest

A.D.C. received fees for lecturing and congress participation from Ultragenyx. M.A. received grants or contracts from Pfizer and Lilly, consulting fees from Amgen, Akcea Therapeutics, Daiichi Sankyo, Ultragenyx, Novartis and Arrowhead, payment or honoraria for lectures, presentations, speakers' bureaus, manuscript writing or educational events from Amgen, Amryt Pharmaceutical, Daiichi Sankyo, Regeneron, Sanofi, Amarin and Ultragenyx. L.D. received a consultancy grant from Amryt, honorary for lecturing from Amryt, Sobi, AuroraBiopharma, Novartis, Amarin, Daiichi‐Sankyo, Bayer, Chiesi, Ultragenyx, Sandoz, honoraria for advisory board from Amarin, and support for attending meetings and/or travel from Daiichi‐Sankyo, Amryt and Chiesi.

## Supporting information


**Figure S1:** Study participants inclusion and attrition flow diagram.
**Figure S2:** Untreated LDL‐C levels in *APOB* variant carriers in the UKBB cohort.
**Figure S3:** 15‐years cumulative incidence of ASCVD events in *APOB* carriers stratified by steatogenic risk factors.
**Table S1:** List of ICD‐9 and ICD‐10 codes used to classify diseases, severity and causes of death in UKBB individuals.

## Data Availability

The data that support the findings of this study are available from UK Biobank. Restrictions apply to the availability of these data, which were used under licence for this study. Data are available from https://www.ukbiobank.ac.uk/enable‐your‐research/apply‐for‐access with the permission of UK Biobank.
