# Supplementary material for: Low Cholesterol due to APOB Variants: Exploring the Balance Between Liver and Cardiovascular Risk
Source: Liver Int. 2026 Jan 19;46(2):e70515. doi: 10.1111/liv.70515 (PMC12813735; doi:10.1111/liv.70515)
Supplement: Supplementary file 1 — Figure S1: Study participants inclusion and attrition flow diagram. Figure S2: Untreated LDL‐C levels in APOB variant carriers in the UKBB cohort. Figure S3: 15‐years cumulative incidence of ASCVD events in APOB carriers stratified by steatogenic risk factors. Table S1: List of ICD‐9 and ICD‐10 codes used to classify diseases, severity and causes of death in UKBB individuals. [file LIV-46-0-s001.docx]

**Supplemental Material**

This appendix has been provided by the Authors to give readers additional information about their work.

Supplement to: Di Costanzo A, Pirona I et al. **Low cholesterol due to *APOB* variants: exploring the balance between liver and cardiovascular risk**

**Supplemental Methods**

**Figure S1.** Study participants inclusion and attrition flow diagram

**Figure S2.** Untreated LDL-C levels in *APOB* variant carriers in the UKBB cohort

**Figure S3.** 15-years cumulative incidence of ASCVD events in *APOB* carriers stratified by steatogenic risk factors

**Table S1.** List of ICD-9 and ICD-10 codes used to classify diseases, severity and causes of death in UKBB individuals

**Supplemental Methods**

Selection of inactivating variants in *APOB*

The overall strategy to identify carriers and non-carriers of inactivating variants in *APOB* is summarized in the **Figure S1**. First, UK BiLEVE Axiom and UK Biobank Axiom Arrays based on 500,000 individuals as well as exomes from 250,000 individuals were used to obtain genetic information of the entire cohort. BCFtools**^13^** and PLINK (<http://pngu.mgh.harvard.edu/purcell/plink/>) **^14^** were used to extract rare variants. The overlap of loss of function (LoF) variants found in individuals with both array and exome was performed using the ‘Pandas’ library (v.2.1.4) from Python (v.3.10.13 - https://github.com/pandas-dev/pandas). Overall, 188 LoF variants in *APOB* were, firstly, selected as being assigned as high impact, high-confidence LoF by the LOFTEE plugin for the Ensembl Variant Effect Predictor (VEP) software (version 107)**^15^** and annotated by using Varsome Premium (v.4.0) (**see Figure S1**). LOFTEE applies a set of filters to identify high-confidence inactivating variants based on predicted impact on the resulting transcript **^16^**.

To restrict the analysis to only those variants that could have a possible protein-lowering effect, we tested the individual effect of all high-impact, high-confidence LoF variants on untreated LDL-C levels. Before doing this, first, we excluded all individuals with reported in-hospital diagnoses of dyslipidemias such as pure hypercholesterolemia, hypertriglyceridemia, mixed hyperlipidemia, and hyperchylomicronemia as well as those with secondary causes of low cholesterol levels, namely intestinal malabsorption, and coeliac disease diagnoses (**Table S1)**. Second, to reduce possible measurement errors, for those having complete lipid data, we excluded all individuals identified as LDL-C outliers based on LDL-C levels below the age- and sex-adjusted 5^th^ percentile (among non-carriers) and above the age and sex-adjusted 95^th^ (among carriers) of the general population **^17^**. Then, the association of *APOB* LoF variants with estimated untreated LDL-C levels were tested by adjusted mixed linear models **^18-19^**. Overall, 32 high-impact, high-confidence LoF variants in *APOB* were extracted and considered as ‘*inactivating*’ as being associated with significantly low plasma LDL-C concentrations at significance levels of *P_adj_*≤0.05 (**Figure S2)**. All individuals carrying *APOB* variants that were not significantly associated with LDL-C level were excluded from the final analysis.

As shown in **Figure S2**, a small number of *APOB* LoF variant carriers (n=14) exhibited LDL-C levels > 160 mg/dL. These individuals were not excluded, as their LDL-C values did not meet the exclusion criteria for statistical outliers based on age- and sex-adjusted population >95th percentile **^17^**, and their inclusion did not affect any of the study results. Therefore, a cohort of 241 *APOB* carriers were retained in the final analyses (see **Figure S1**)’.

*Assessment of liver and ASCVD outcomes*

In-hospital records and cause-of-death registry (Fields 41270, 41271, 40001 and 40002) were used to define liver and ASCVD outcomes and to estimate primary and secondary causes of death. For primary liver outcomes, we considered the ICD-9 and ICD-10 codes listed in the **Table S1**. Chronic liver disease (CLD) outcome was defined as follows:

- **chronic liver disease** (CLD) cases were defined as those patients meeting non-alcoholic fatty liver (K76.0), nonalcoholic steatohepatitis (K75.8), liver disease unspecified (K76.9), alcoholic fatty liver (K70.0), alcoholic liver disease unspecified (K70.9), alcoholic cirrhosis (K70.3), unspecified cirrhosis of liver (K74.6), esophageal varices (I85.0 - I85.9 - 4560 - 4561), portal hypertension (K76.6), liver cell carcinoma (C220) and liver cancer (C22.9) criteria.

As reported previously **^21^**, individuals with a concurrent diagnosis of chronic (viral) hepatitis or acute hepatitis C (n=175) were excluded from the analysis of liver outcomes **(Table S1)**. Individuals not having in-hospital ICD-9 and ICD-10 liver disease diagnoses but recorded as deceased for causes associated with liver diseases were also counted as cases, as previously reported (n=2 among *APOB* carriers and n=418 among non-carriers) **^21^**. To avoid overestimating the number of diagnoses, only the first event was selected for each individual during follow-up. Using these criteria, 5,967 individuals were considered as affected by liver disease (cases) and 404,820 individuals as free from liver disease (controls).

The ICD-9 and ICD-10 codes used to classify individuals affected by atherosclerotic cardiovascular diseases (ASCVD) are listed in the **Table S1**. A composite ‘ASCVD’ outcome was used to identify those meeting any of the criteria below:

-unstable angina (I20.0), other forms of angina pectoris (I20.8), angina pectoris unspecified (I20.9), angina pectoris (413 - 4139), acute myocardial infarction (I21 all - 410 - 4109), subsequent myocardial infarction (I22 all), certain current complications following acute myocardial infarction (I23 all), other acute ischaemic heart diseases (I24 all), other acute and subacute forms of ischaemic heart disease (411 - 4119), old myocardial infarction (412- 4129), chronic ischaemic heart disease (I25 all), other forms of chronic ischaemic heart disease (410 - 414 - 4140 - 4141 - 4148 - 4149), ill-defined descriptions and complications of heart disease (42979), cardiovascular disease, unspecified (I51.6), cerebrovascular diseases and stroke: cerebral infarction (I63 all), stroke not specified (I64), other specified cerebrovascular diseases (I67.8) and cerebrovascular disease unspecified (I67.9), occlusion of cerebral arteries (434 - 4349), acute, but ill-defined, cerebrovascular disease (436 - 4369). As above, individuals not having in-hospital ASCVD disease diagnosis but recorded as deceased for causes associated with cardiovascular diseases were also counted as cases (n=1 among *APOB* carriers and n=1,377 among non-carriers). As above, only the first occurring ASCVD event was selected in each individual during follow-up. Among 410,962 individuals, n=30,797 were classified with (cases) and n=380,165 without ASCVD events (controls).

**Figure S1. Study participant inclusion and attrition flow diagram**


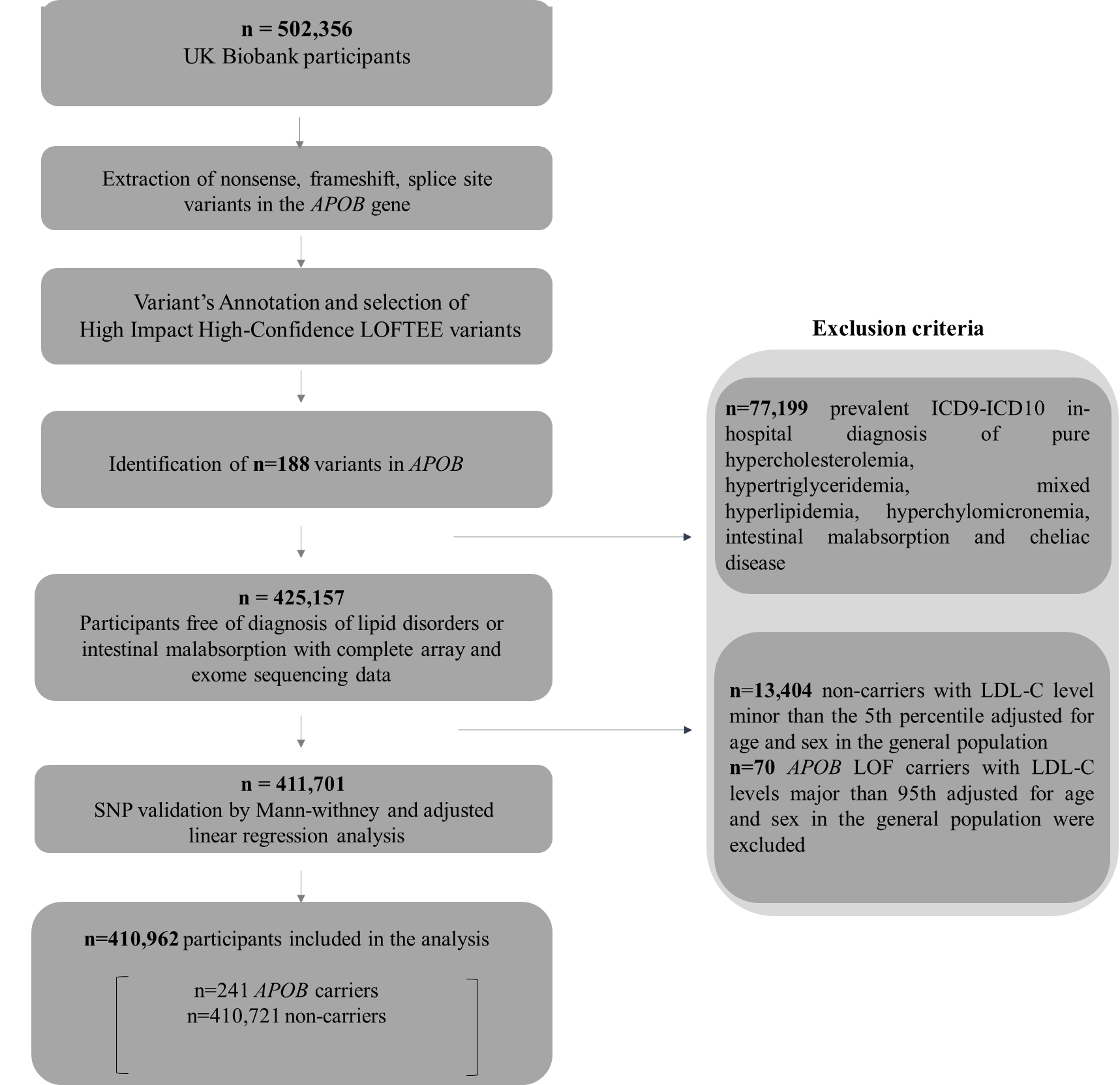


**Figure S2. Untreated LDL-C levels in *APOB* variant carriers in the UKBB cohort**

***
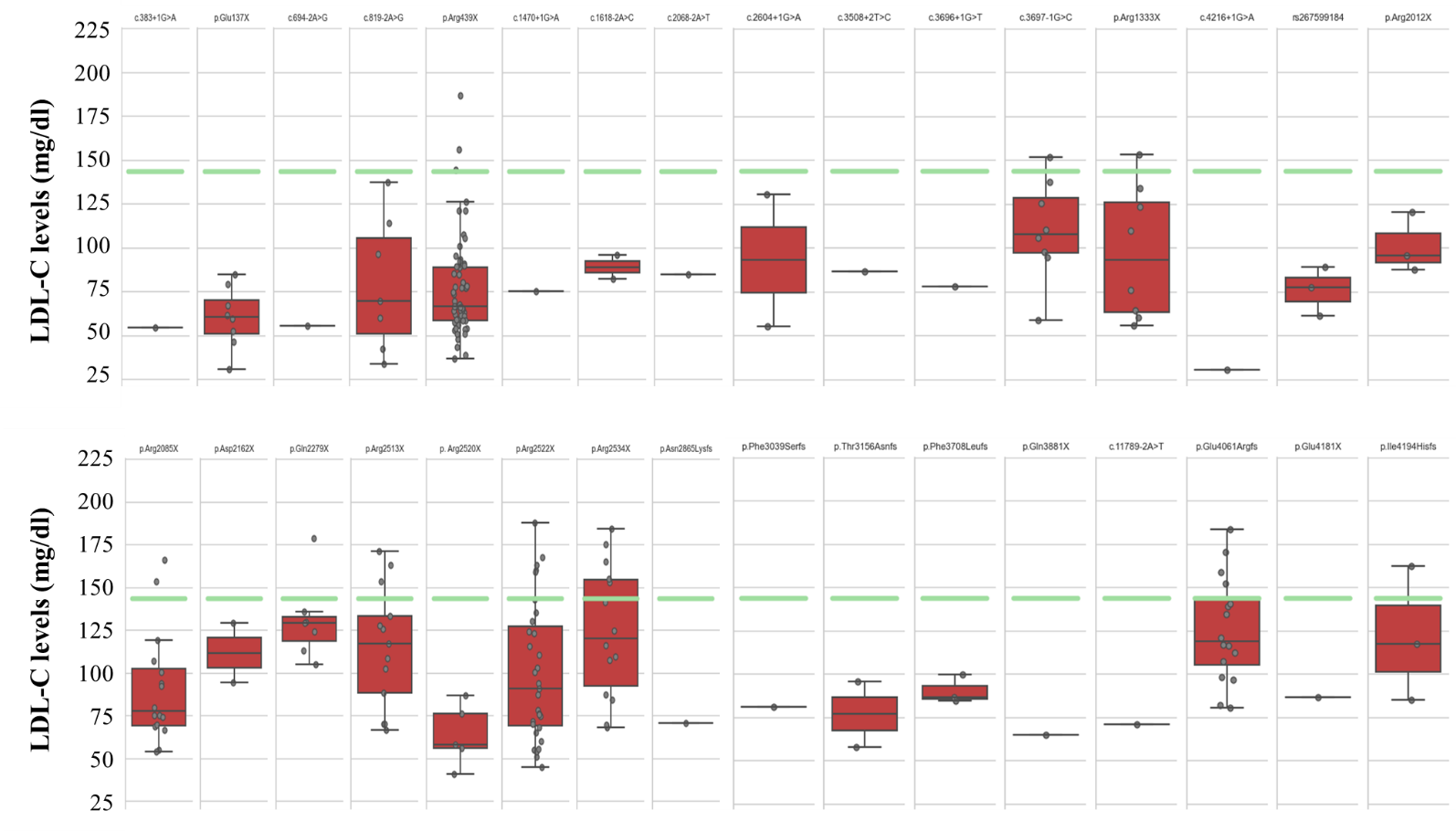
***

**Figure Legend:** The green line indicates the estimated untreated LDL-C median values of non-carriers, which correspond to 140.37 (121.49-161.45) mg/dl. Individual protein-sequence variants are listed in order of their position in the protein. HGVS nomenclature is relative to the NM_000384.3 for *APOB* gene. Variant position numbers are reported in the context of the reference human genome build, hg19. The nomenclature of each variant was verified by using Varsome premium (<https://landing.varsome.com/varsome-premium>). We accounted for the effect of lipid-lowering therapy in participants reporting such use at the time of lipid measurement by dividing the measured LDL cholesterol by 0.7 **^6^**. The analysis was restricted to individuals with complete lipid measurements.

**Figure S3.** **15-years cumulative incidence of ASCVD events in *APO*B carriers stratified by steatogenic risk factors**


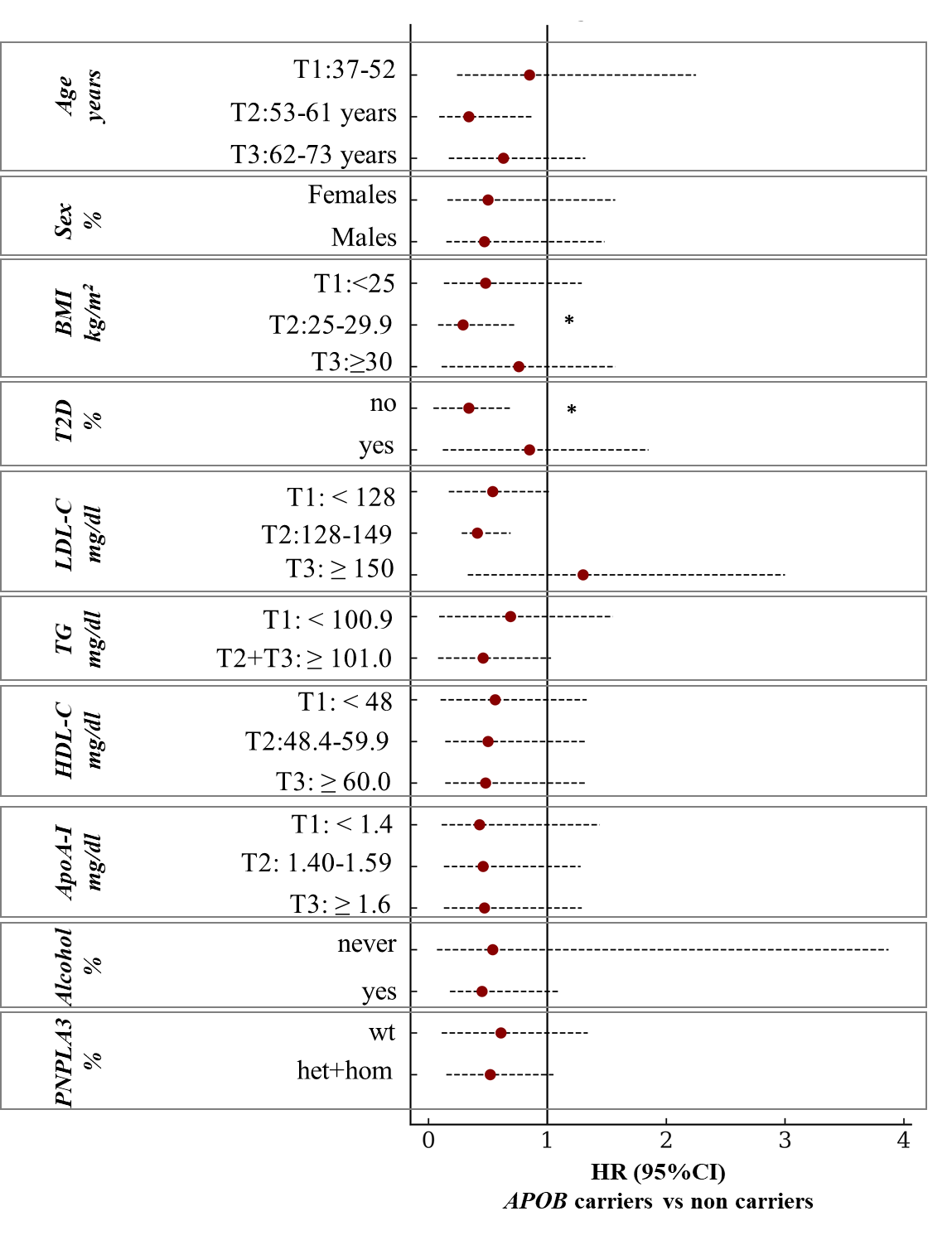


**Figure Legend:** The figure shows the cumulative incidence during 15-years of follow up for ASCVD in *APOB* carrier’s versus non-carriers stratified by steatogenic risk factors. Lipid-related strata were defined as follows: LDL-C tertiles: : T1 < 128 mg/dL; T2 = 128 – 149 mg/dL; T3 ≥ 150 mg/dL; HDL-C tertiles: T1 < 48.0 mg/dL; T2 = 48.4–59.9 mg/dL; T3 ≥ 60.0 mg/dL; Triglycerides (TG): T1 < 100.9 mg/dL; T2+T3 ≥ 101.0 mg/dL; ApoA-I tertiles: T1 < 1.4 mg/dL; T2 = 1.40–1.59 mg/dL; T3 ≥ 1.60 mg/dL.

HRs and 95% CIs were based on Cox regression analyses performed with Firth penalization. * Firth-penalized P ≤ 0.05 for comparison between carriers and non-carriers without diabetes. Only statistically significant P values are reported.

**Table S1. List of ICD-9 and ICD-10 codes used to classify diseases and causes of death in UKBB individuals**

| **HYPERTENSION** | Essential (primary) hypertension (I10), secondary hypertension (I15) |
| --- | --- |
| **DIABETES** | Insulin-dependent diabetes mellitus (E10 all), non-insulin-dependent diabetes mellitus (E11 all), malnutrition-related diabetes mellitus (E12 all), other specified diabetes mellitus (E13 all), unspecified diabetes mellitus (E14 all), glomerular disorders in diabetes mellitus (N08.3), pre-existing diabetes mellitus, insulin-dependent (O24.0), pre-existing diabetes mellitus, noninsulin-dependent (O24.1), pre-existing malnutrition-related diabetes mellitus (O24.2), pre-existing malnutrition-related diabetes mellitus (O24.3), diabetes mellitus (250 all), diabetes with ketoacidosis (25010 all), diabetes with coma (2502 all), diabetes with renal manifestations (2503), diabetes with ophthalmic manifestations (2504), diabetes with neurological manifestations (2505), diabetes with unspecified complications (adult-onset type) (25099). |
| **INTESTINAL MALABSORPTION** | Coeliac disease (K90.0 - 5790), malabsorption due to intolerance, not elsewhere classified (K90.4), other intestinal malabsorption (K90.8), intestinal malabsorption, unspecified (K90.9 - 5799). |
| **LIPID AND LIPOPROTEIN DISORDERS** | Pure hypercholesterolemia (E78.0 - 2720), pure hypertriglyceridemia (E78.1 - 2721), mixed hyperlipidemia (E78.2 - 2722), hyperchylomicronemia (E78.3) , other hyperlipidemia (E78.4 - 2724), hyperlipidemia, unspecified (E78.5). |
| **LIVER OUTCOME** | |
| **HEPATITIS B/C** | Hepatitis B or C (B18.0 - B18.1 - B18.2 - B18.8 - B18.9), acute viral hepatitis (B17.1)  acute hepatitis C (B17.9) |
| **NAFLD/NASH** | Non-alcoholic fatty liver (K76.0), other specified inflammatory liver diseases including non-alcoholic steatohepatitis (K75.8) |
| **LIVER CIRRHOSIS (NON-ALCOHOLIC)** | Unspecified cirrhosis of liver (K74.6, 5715), esophageal varices (I85.0, I85.9, 4560, 4561), portal hypertension (K76.6, 5723). |
| **ANY LIVER CIRRHOSIS** | Alcoholic cirrhosis (K70.3, 5712,5713), unspecified cirrhosis of liver (K74.6, 5715), esophageal varices (I85.0,I85.9, 4560, 4561), portal hypertension (K76.6, 5723). |
| **LIVER CELL CARCINOMA** | Liver cell carcinoma (C22.0), unspecified liver cancer (C22.9). |
| **ANY LIVER DISEASE (ALCOHOLIC)** | Alcoholic fatty liver (K70.0, K70.1, K70.2, K70.4, K70.9), Alcoholic cirrhosis (K70.3, 5712,5713) |
| **ANY LIVER DISEASE (NON-ALCOHOLIC)** | Non-alcoholic fatty liver (K76.0), nonalcoholic steatohepatitis (K75.8), liver disease unspecified (K76.9), unspecified cirrhosis of liver (K74.6), esophageal varices (I85.0 - I85.9 - 4560 - 4561), portal hypertension (K76.6). |
| **ASCVD OUTCOME** | |
| **ISCHEMIC HEART DISEASES** | Unstable angina (I20.0), other forms of angina pectoris (I20.8), angina pectoris unspecified (I20.9), angina pectoris (413 - 4139). |
| **ACUTE AND SUBSEQUENT MYOCARDIAL INFARCTION** | Acute myocardial infarction (I21 all - 410 - 4109), subsequent myocardial infarction (I22 all), certain current complications following acute myocardial infarction (I23 all), other acute ischaemic heart diseases (I24 all), other acute and subacute forms of ischaemic heart disease (411 - 4119), old myocardial infarction (412- 4129). |
| **CHRONIC ISCHAEMIC HEART DISEASE** | Chronic ischaemic heart disease (I25 all), other forms of chronic ischaemic heart disease (410 - 414 - 4140 - 4141 - 4148 - 4149), ill-defined descriptions and complications of heart disease (42979). |
| **COMPLICATIONS AND ILL-DEFINED DESCRIPTIONS OF HEART DISEASE** | Cardiovascular disease, unspecified (I51.6), cerebrovascular diseases and stroke: cerebral infarction (I63 all), stroke not specified (I64), other specified cerebrovascular diseases (I67.8), cerebrovascular disease unspecified (I67.9), occlusion of cerebral arteries (434 - 4349), acute, but ill-defined, cerebrovascular disease (436 - 4369) |
